# Neurodiversity: bridging the gap in UK's GP training for a more inclusive future

**DOI:** 10.3389/fmed.2025.1587283

**Published:** 2025-08-22

**Authors:** Waseem Jerjes, Azeem Majeed

**Affiliations:** Department of Primary Care and Public Health, Faculty of Medicine, Imperial College London, London, United Kingdom

**Keywords:** neurodiversity, GP training, inclusive education, mentorship, autism and ADHD

## Introduction

General practice (GP) in the UK has long accommodated the needs of the diverse population it serves. Increasingly acknowledged is another form of this diversity: the natural variation of cognitive functioning we now speak of as neurodiversity—conditions such as autism, attention deficit hyperactivity disorder (ADHD), and dyslexia. These are not deficits to be remediated but rather represent differing ways of processing information, being social, and responding to the environment. With greater understanding of neurodiversity comes the need to specifically consider how GP training uniquely impacts neurodivergent learners. GP training encompasses varied clinical, interpersonal, and administrative competencies, often requiring rapid switching between tasks, sensory-rich environments, and extensive patient interactions. These elements present unique challenges and opportunities for neurodiverse trainees, making GP training an ideal context for targeted, inclusive educational strategies.

Based on NHS statistics, roughly 1 individual out of 100 is autistic, and up to 2.5 million individuals across England have the potential to have ADHD, including up to 740,000 children (1, 2). However, current GP training processes often do not adequately incorporate or address the specific learning needs of neurodivergent trainees. Where support is provided, it is generally reactive instead of proactive, and neurodivergent doctors consistently face unnecessary barriers to their wellbeing and professional growth.

As clinicians and educators engaged in postgraduate GP training and inclusion work, we draw on our professional experiences and the voices of neurodiverse colleagues and trainees to propose practical reforms grounded in real-world contexts. In this opinion paper, we explore the gaps in the NHS GP training system with regard to neurodiversity and propose ways to foster a more inclusive and supportive environment for neurodiverse GP trainees. Beyond fairness and trainee support, inclusive GP training has the potential to improve patient care by cultivating a diverse workforce better equipped to understand and address the varied needs of neurodiverse patient populations. By ensuring that GP trainees reflect the diversity of the communities they serve, the healthcare system becomes more responsive, empathetic, and effective.

## The need for a proactive approach

Despite growing awareness of neurodiversity, the UK, GP training system remains predominantly reactive in its approach (3, 4). Trainees with cognitive differences often receive support only after a significant event, such as exam failure or a major performance issue. This delay in support undermines their confidence and potential for development, leaving them to struggle unless timely intervention is offered. By the time support is given, many trainees may have already experienced unnecessary stress, reduced confidence, and delayed career progression.

A key challenge is that neurodiverse traits are not always easy to recognize. GP trainers may not always have the knowledge or training to identify these traits, which can manifest subtly and be mistaken for other issues (5). For example, a trainee with ADHD might be seen as disorganized or lacking in time management skills, while an autistic trainee's attention to detail could be seen as a personality trait (3). Without greater understanding of neurodiverse conditions, trainers can inadvertently misinterpret trainee behaviors, negatively impacting the learning process. This emphasizes the need to embrace a neurodiversity-informed mindset—one that treats cognitive variations as plausible explanations of observed behaviors, even in the absence of formal diagnosis or disclosure.

Recent literature further highlights complexities around disclosure, noting that neurodiverse trainees often hesitate to disclose their needs due to stigma and fear of negative professional repercussions (3). Consequently, proactive educational frameworks must move beyond the prevailing medical-model perspective, which seeks to “fix” the learner by focusing primarily on individual deficits and remediation strategies, toward adopting a biopsychosocial model that considers broader environmental, social, and psychological factors to enhance compatibility between trainees and their learning contexts (6, 7). Such a model accommodates the tension between neurodivergent identity and professional identity by focusing on systemic adjustments, personalized support, and reducing reliance on generic accommodations, thereby fostering a more inclusive, empowering, and psychologically safe training experience.

## Addressing systemic shortcomings

The traditional GP training curriculum strongly relies upon rigid instructional formats such as lectures and constrained timetables, which may not suit all learning styles—particularly neurodiverse trainees (2, 4). Learners with autism, ADHD, or sensory sensitivity may find these formats uncomfortable, potentially reducing their productivity and performance quality in stimulating environments, such as busy GP surgeries or distracting study rooms (6–9). Additionally, traditional examinations that emphasize rote memory over thought analysis can put neurodiverse learners at a disadvantage, who may find their strengths lie with practical, thoughtful, or system-oriented approaches (2, 6).

In educational settings outside of medicine, early identification and support for neurodiverse students leads to better academic outcomes and lower stress levels (7). The same is true in workplaces where accommodations are made to meet the unique needs of neurodiverse employees (8–12). By integrating similar proactive measures into GP training, we can create an environment that nurtures neurodiverse talents and allows these trainees to reach their full potential.

## Training GP trainers: a key intervention

For all such changes to take effect, GP trainers need to be educated on the topic of neurodiversity. The goal of neurodiversity education for GP trainers should be to enable them to effectively identify, understand, and support neurodiverse trainees. To achieve this, education should move beyond broad awareness levels and incorporate structured training modules, including interactive workshops, scenario-based exercises, role-playing activities, and reflective group discussions. For example, training could be delivered through blended learning formats—combining online modules covering theoretical understanding of neurodiversity, in-person or virtual workshops for practical scenario-based exercises, role-play to enhance trainer empathy, and reflective group discussions to address unconscious bias. Such structured and multimodal delivery ensures trainers not only gain knowledge about neurodiverse conditions but also acquire practical skills for identifying and effectively supporting neurodiverse trainees (7, 13, 14). Incidental unconscious bias and diagnostic masking, which can get in the way of identifying the neurodiversity of trainees, should also be addressed (14). With such focused education, GP educators would better understand the strengths and difficulties of their neurodiverse trainees and provide specific support accordingly.

Educators trained in neurodiversity are recommended to better support neurodiverse learners by employing autism-informed and Universal Design strategies, creating more inclusive educational environments (15). UK GP trainers at the moment complete formal accreditation processes to retain their position. Neurodiversity education is something that may easily become part of this framework through integration as an innate competency module or elective CPD module. Embedding education around neurodiversity through existing trainee development programs makes the system more responsive to the individual learner's needs and inclusive.

## Flexible training programmes and individual learning plans

One of the most effective ways to support neurodiverse GP trainees is through flexible, individualized learning programmes (Table 1) (10–12). For example, trainees with ADHD might benefit from organizing shorter, more frequent self-directed study sessions, with trainers offering flexibility and encouragement to accommodate these patterns, while those with sensory sensitivities may prefer quiet study spaces or environments with softer lighting. Trainers should remain aware of these preferences and be flexible where possible to support them. Assistive technologies, such as noise-canceling headphones or specialized software, are often already used by neurodiverse trainees; trainers should respect and support their use during placements and assessments. Implementing individual learning plans and proactively adjusting educational environments are recommended strategies to reduce barriers, improve accessibility, and create inclusive learning conditions for neurodiverse learners (7, 16, 17), suggesting that these strategies could have a profound impact on GP trainees as well.

**Table 1:** Practical recommendations to support neurodiverse trainees in UK GP training.

**Theme**	**Specific recommendation**	**Who is responsible**	**Rationale/expected benefit**
Training flexibility	Offer ILPs for trainees.	GP trainers/TPDs	Tailors support to the individual profile of each neurodiverse trainee.
Learning environment	Provide quiet study areas, adjust lighting, and minimize sensory overload.	Training practices	Reduces stress and enhances concentration for trainees with sensory needs.
Assessment methods	Incorporate practical assessments, portfolios, and case-based discussions.	Training programme designers	Allows neurodiverse trainees to showcase their strengths beyond rote exams.
Trainer development	Embed neurodiversity awareness into educator CPD.	Deaneries/Educator faculties	Ensures trainers can support trainees proactively and confidently.
Peer and mentor support	Facilitate neurodiversity-informed mentorship and peer discussion groups.	VTS scheme leads/Practices	Reduces isolation, shares coping strategies, and boosts morale.
Workplace adjustments	Offer assistive tools (e.g., noise-canceling headphones), flexible rotas, and low-stimulation clinics.	GP practices	Supports sensory regulation and working style preferences.
Policy and advocacy	Include neurodiversity in trainee surveys, policies, and ARCP support pathways.	HEE/NHS Education bodies	Drives systemic change and supports long-term inclusion.

ILPs, Individual Learning Plans; GP, General Practice; TPDs, Training Programme Directors; CPD, Continuing Professional Development; VTS, Vocational Training Scheme; HEE, Health Education England; NHS, National Health Service; ARCP, Annual Review of Competence Progression.

Diversifying the types of assessments is also important. Rather than depending on conventional exams, GP training might involve practical demonstrations, portfolio assessments, and application to actual clinical settings to evaluate the more varied range of skills and abilities (18). These alternative assessments would permit the strengths of neurodivergent trainees—e.g., recognition of patterns, innovative solution-finding, and increased attention to detail—to come through, which may elude conventional written exams under time constraint. Additionally, alternative formats can alleviate cognitive load and worries, so trainees can engage more genuinely with their education (7, 13). By integrating more inclusive strategies, the evaluation becomes a more accurate reflection of clinical proficiency rather than test-taking skill.

We recognize that the implementation of flexible, individualized approaches can face practical challenges, including variability in trainer readiness, concerns around equitable standardization of assessments, and resource limitations. To mitigate these challenges, training frameworks should incorporate principles of universal design for learning (UDL), creating environments that inherently accommodate diverse cognitive profiles, thereby reducing the need for individualized adjustments (7, 19). Furthermore, adopting an explicitly strengths-based approach—focusing on leveraging unique cognitive abilities rather than merely addressing deficits—can foster more inclusive, empowering, and resilient learning environments for all GP trainees (6, 10, 11).

Meanwhile, neurodiverse trainees should be equipped with the appropriate tools and guidance to enable them to advocate for their needs for reasonable adjustments and better communicate their own learning requirements (16).

## Mentorship and peer support

Mentorship plays an essential role in the professional development of all GP trainees, but it is especially important for neurodiverse individuals. Many neurodiverse trainees struggle to find mentors who understand their experiences and challenges. Within GP training, mentorship is often informal or practice-based; however, these structures rarely account for cognitive diversity or neurodivergent communication styles (20). A revision of mentorship programs is needed—this could include mentor-mentee matching based on shared communication preferences, embedding neurodiversity training into existing mentor development schemes, and establishing opt-in mentorship structures that respect voluntary disclosure (21).

Peer support groups may also support formal mentoring through the provision of safe psychological space to support collective learning, validation, and mutual support—all the more valuable where such settings can otherwise become isolating for neurodivergent trainees (18). Neurodiversity-trained mentors, in their mentoring, may offer guidance sensitive to the special needs of their mentees, helping to create more positive and productive learning settings. Well-organized and inclusive mentoring has been linked to enhanced educational attainment, improved confidence, and lower stress among neurodiverse learners. Recent studies and expert recommendations highlight the potential benefits of mentorship tailored to neurodiverse learners, suggesting that structured mentorship may reduce stress and anxiety and support professional and academic success among neurodivergent populations (14, 15).

## Creating sensory-friendly GP practices

There are also ways GP practices can be made more neurodiversity-friendly. Basic physical modifications—such as reducing sensory distress through softer lighting, minimizing background noise, or the provision of noise-canceling headphones—have been found to enhance comfort and concentration for those who are neurodivergent in the working environment (19). Further, an understanding and supportive workplace culture is also needed. In our experience, structured routines and explicit expectations are important facilitators of success for autistic professionals, particularly in healthcare and education settings.

Neurodiverse trainees can perform well at working with patients on an individual basis but struggle with noisy, distracting group situations. Designing an environment to respect such inclinations—including forward-thinking adjustments and inclusive team cultures—can powerfully support trainee performance and job satisfaction. Additionally, sensory-friendly GP practices not only benefit neurodiverse trainees but also enhance care experiences for neurodiverse patients, creating an inclusive healthcare setting for all (22). By taking action to modify both environmental and culture-based factors, GP practices can enable neurodiverse trainees to thrive, with the end result being an enhanced workforce and better patient care.

## Advocating for systemic change

More than individual efforts are needed to create inclusivity in GP education. Advocacy needs to run through all tiers of the NHS to increase awareness of and reduce the stigma around neurodiversity. Recent developments, including the introduction of the Oliver McGowan Mandatory Training on Learning Disability and Autism, have been important steps toward the education of healthcare professionals on neurodivergent states. This mandatory training, laid down by the law for NHS staff, is helping to improve understanding and support for neurodivergent colleagues and patients (23).

Conferencing, publications, and disseminating success stories of neurodiverse GP trainees are key to reframing attitudes and inspiring more trainees to come forward and ask for the support they need. The Royal College of General Practitioners has, for example, run events on the subject of neurodiversity and medicine, giving space to those who are neurodivergent to tell their stories and speak up for change to the system. These stories not only personalize the difficulties encountered but also identify the specific strengths that people who are neurodiverse bring to the medical profession (24).

Policymakers and healthcare institutions need to play their part in initiating such changes, where their institutions identify and ensure the recognition of neurodiversity and support the neurodivergent members at all levels of their medical education. The NHS Equality, Diversity, and Inclusion Improvement Plan stresses the need to establish greater inclusivity for all healthcare staff members, including the neurodivergent ones. Integrating such values into the institutions' policies and training programs is the goal of the NHS to develop an inclusive culture where diversity is promoted and all the trainees are able to succeed (25).

This advocacy should be informed and guided by existing UK legal and professional frameworks, notably the Equality Act 2010, which mandates reasonable adjustments and prohibits discrimination based on disability, including neurodivergent conditions. Additionally, relevant guidance from the General Medical Council (GMC), such as the “Welcomed and Valued” guidelines, explicitly emphasizes the responsibilities of medical education bodies to provide inclusive education and reasonable adjustments for neurodiverse trainees (26–28).

## Conclusion

The UK's system of GP training has taken positive steps toward being inclusive, but it has further to travel to meet the varied needs of neurodivergent trainees. True inclusivity demands more than diverse methods of testing and adequately trained mentors, but also the reform of both the GP trainee curriculum and the GP trainers' educational curriculum, infusing awareness of neurodiversity and inclusive learning practices at all levels. This needs to move beyond the realms of mentorship to GP trainers and faculty members—the central actors who define the shape of training climates—who themselves have to become competent to recognize, support, and advocate for numerous cognitive profiles. Neurodiversity is not, however, an immutable category; every individual has their own unique combination of characteristics, strengths, and weaknesses.

Thus, GP trainers' educational programmes should not merely prepare trainers to deliver standardized instruction but instead develop their skills in questioning, sensitivity, and reflective practice, enabling them to work effectively with neurodiverse trainees. By accepting the entire range of cognitive diversity among its physicians and adjusting the systems accordingly, UK general practice can develop a model where all trainees—across all neurotypes—are enabled to flourish and excel.
